# What Do Public Health Graduates Do and Where Do They Go? An Analysis of Job Destinations and Mismatch in Australian Public Health Graduates

**DOI:** 10.3390/ijerph18147504

**Published:** 2021-07-14

**Authors:** Rory David Watts, Devin C. Bowles, Colleen Fisher, Ian W. Li

**Affiliations:** 1 School of Population and Global Health, The University of Western Australia, Crawley, WA 6009, Australia; colleen.fisher@uwa.edu.au (C.F.); rorrry.watts@uwa.edu.auee (I.W.L.); 2 National Centre for Epidemiology & Population Health, Australian National University, Canberra, ACT 0200, Australia; rory.watts@uwa.edu.auee

**Keywords:** public health, population health, workforce, training, employment, professionalism, interdisciplinarity

## Abstract

Background: It is not well understood what occupations public health graduates have after graduation, nor is it well known whether their education provides them with the relevant knowledge and skills to feel well matched to their occupations. Furthermore, it is commonly presumed that public health graduates work in government, and investments in education would bolster this workforce. Methods: We aimed to describe the common occupations of Australian public health graduates, describe the heterogeneity of graduate destinations, describe the level of mismatch that graduates report, and compare these results with other fields of study. We used eight years of Australian graduate survey data (2008–2015) from the Graduate Destinations Survey, examining outcomes data from 8900 public health graduates from four levels of education. We compared occupation and industry heterogeneity, and level of occupational mismatch between public health graduates, and graduates from other fields of education. Results: Public health graduates report having a broad set of occupations in a broad set of industries after graduation, and this breadth is dissimilar to most health degrees. Furthermore, public health graduates tend to have average or lower-than-average rates of mismatch. Conclusions: Despite going into a broad set of occupations and industries, graduates from public health tend to report being well prepared given their education. Given that both occupation and industry outcomes are heterogeneous for graduates, an investment in public health education does not guarantee an increase in the governmental public health workforce.

## 1. Introduction

Fields of study may have a strong relationship with occupations. Strong relationships are common, and may be regulated, such as nursing, medicine, or psychology. Others are intended to be concordant, where education prepares an individual for an occupation, but does not guarantee it [1,2]. Weak relationships are common too, but interpretation of these relationships is more difficult. One may perceive a weak relationship as a boon, where the student has such flexible skills that they are capable of work in many occupations and industries. On the other hand, a weak relationship may imply that students have been trained in a way which is not well recognized by any employer.

Due to the COVID-19 pandemic, we have seen simultaneous calls to strengthen the ‘public health workforce’ and to increase funding into public health education [3,4]. These calls are making two claims. The first claim is that we should bolster our workforce now, and strengthen it in the future, which is hard to rebut. The second claim is that public health education leads to public health occupations. However, the set of public health occupations which are needed are not often clearly articulated, and Australian data emphasizes a breadth of positions for graduates, many of which are outside the government. For example, Rotem et al. found that Master of Public Health (MPH) students between 1989 and 1993 had sixteen different occupational backgrounds (many clinical) [5], and only one in six students were looking for a different career. In 2014, Li and Awofeso found that public health postgraduates became managers, health professionals, science professionals, business professionals and many other occupations, and nearly half did not have occupations in the health sector [6]. Houghton, Braunack-Mayer and Hiller interviewed graduates from Adelaide University’s Bachelor of Health Sciences from 1992 to 1999 [7], finding that whilst the majority (59%) worked in ‘public health’, they were ‘doing so in a wide range of roles, reflecting the diversity of the public health workforce’. In 2019, Watts et al. found that public health job advertisements are distributed among government, not-for-profit, academia, and the private sector, whilst also having a great deal of occupational variance [8].

Occupational variance is not only the case in Australia. Plepys et al. (2021) found that employed graduates from the United States entered health care, corporations, academia, government, non-profit, ranging from 27% in health care, to 12% in non-profits [9]. Krasna et al. (2021), found that postgraduate public health students from Columbia University between 2012 and 2016 went into non-profit, government, and private sectors [10]. Importantly, these paths had changed over time, with an increasing tendency for graduates to enter the private sector, a decreasing tendency to enter the non-profit sector, and a steady entrance into government; approximately 12% of graduates. The authors noted that while most discussions are about governmental public health work, this is not the common trajectory of their studied cohorts. Occupational variance from public health graduates has also been observed in South Africa and Canada, but to lesser extents. MPH graduates from the University of KwaZulu-Natal in South Africa were surveyed in 2013, and 63% of respondents reported working in government, whereas 22% were in academia, 6% were in ‘research’ and 6% were in a non-governmental organisation [11]. In Canada, MPH graduates of the University of Guelph were surveyed in 2013 [12], finding 60% of respondents were employed in the public sector. Encouragingly, of those employed in the public sector, 71% found employment in less than 3 months (data were not available for those outside of the public sector). Indeed, the heterogeneity of occupational outcomes are likely related to the country of investigation [13], but currently, evidence often focuses on cohorts from individual universities, and graduates from MPH degrees.

The existing literature also suggests that the educational origins of public health workers are not solely from public health either. In Australia, Rotem et al. surveyed public health postgraduates who were performing public health functions, finding that only 14% of them had public health qualifications. This has been found in other countries too. For example, approximately 3% of the public health workforce in New Zealand have an MPH [14], as do one-third of workers in Switzerland [15], and approximately half of public health workers in some governmental US agencies [16]. Therefore, one should not expect, a posteriori, that there is a clear, predictable policy outcome from strengthening public health education in Australia.

However, there are some good reasons to explore the relationship between people who receive public health education and their occupations. Firstly, much of the data from Australia are dated, and it is worth updating this knowledge. Secondly, we do not have a good sense of what occupations graduates actually have, whether these differ based on level of education, and whether graduates feel their education has prepared them well for these occupations. This knowledge may help a student invest their time and money wisely, and may help education providers better articulate the outcomes of their graduates. For example, is a public health degree more similar to a nursing degree, with a clear set of occupational destinations? Or is it more similar to a business degree, where graduates can apply their skills in a variety of domains? Should one expect a clear career path, a set of transferable skills, both, or neither?

In this study, we sought to answer these questions by characterizing the relationship between public health education (at undergraduate and postgraduate levels) and occupations, by answering the following questions:

1. What occupations do graduates have?
  a. What are the most common occupations that graduates of public health have?
  b. Is this set of occupations more or less heterogeneous than other fields of study (e.g., business, engineering, nursing)?
2. Do graduates feel mismatched to their occupations?
  a. To what occupations are graduates least mismatched?
  b. What proportion of graduates are mismatched, and how does this compare to other fields of study?
3. How well does public health education prepare graduates for their occupation?
  a Are there occupations where public health graduates have the lowest mismatch?
  b. How do these findings compare to other fields of study?

## 2. Materials and Methods

### 2.1. Data Sources

To answer our research questions, we used cross-sectional graduate outcomes data from the Australian Graduate Destinations Survey (GDS) from 2008 to 2015 [17]. The Graduate Destinations Survey (GDS) was a component of the Australian Graduate Survey, a national research project that collected data on the post-study activities of graduates. The sample population of the GDS was all graduates who completed a qualification in an Australian university, who are surveyed between four to six months after graduation by their institution of graduation. From 2016 onward, cross-sectional surveys have been redesigned and redistributed as the Graduate Outcomes Survey (GOS) and it is administered by Quality Indicators for Learning and Teaching (QILT). Due to the inability to harmonize questions regarding mismatch between GOS and GDS (see Section 2.4), we did not use data after 2015.

### 2.2. Defining Graduates and Occupations

We defined our study cohorts based on the field of education and the degree level of the graduates. The field of education was coded in the GDS using the Australian Standard Classification of Education ASCED [18], the Australian Bureau of Statistics’ classification system of higher education fields. The ASCED classifies courses by a hierarchical coding system. For example: the two-digit code 06 indicates health, the four-digit code 0613 indicates public health, and the most refined six-digit classification 061303 is environmental health. Within the ASCED, we classified public health as either 61300 or 61399, which correspond to ‘Public health not elsewhere classified’. We chose not to include other classifications within public health (e.g., environmental health), as we were interested in capturing graduates from ‘broad public health degrees’, which were best represented by these two codes. We note that a previous Australian study found that 64% of all postgraduate public health degrees were classified as 61300 or 61399, the majority of which were considered ‘broad public health degrees’ such as the Master of Public Health [19].

We were able to analyze data for four qualification levels of graduates, which were aggregated in the GDS as follows: Postgraduate Diploma or Certificate, ‘Bachelor’s Degree’, Masters by Coursework, and PhD or Doctorate by Coursework. We excluded other levels (e.g., ‘Master’s by Research’), as there were very few graduates in these categories for both public health graduates, and for graduates of other fields, making robust comparisons between groups difficult. Note that ‘Bachelor’s Degree’ was aggregated in the GDS from the following levels: Bachelor (Pass) Degree, Bachelor (Honours) Degree, Undergraduate Diploma, and Graduate Entry Bachelor Degree. Bachelor (Pass) Degree accounted for the vast majority of Bachelor’s entries, with Bachelor (Honours), Undergraduate Diploma and Graduate Entry Bachelor Degree accounting for less than 8.5% of the cohort graduates (data not shown).

Occupations were categorized in the GDS using the Australian and New Zealand Standard Classification of Occupations (ANZSCO) [20], as this is the coding scheme used by the GDS from 2008 onward. The GDS also categorized industries using the Australian and New Zealand Standard Industrial Classification (ANZSIC) [21], and we used this when considering different industries which graduates may enter. Furthermore, we aggregated all medical, midwifery and nursing occupations into “Midwifery and Nursing Professionals” and “Medical Practitioners”, as we felt these were more suited to our research purposes than more disaggregated occupations (e.g., than considering psychiatrists and anaesthetists separately).

For some research questions, we categorized some occupations as ‘clinical occupations’. This category was created using information provided by ANZSCO, which indicates “Registration or licensing is required.” As broad public health degrees, such as the MPH, do not provide a pathway to clinical licensing, we assumed that graduates must have received prior training in that profession. For example, if an MPH graduate listed their occupation as physiotherapy, we concluded that graduate must have previously studied physiotherapy.

### 2.3. Comparing Heterogeneity of Occupations and Industry

Research question 1b involved comparing the heterogeneity of occupations and industries between graduates from different fields of study. To make this comparison, we used the Herfindahl–Hirschman Index (HHI) [22]. The HHI is a measure of the how distributed a group is amongst a set of states. In our case, the group refers to the graduates from a certain field (e.g., public health) and the set of states are the occupations or industries that graduates have. This is formally defined below:

(1) $$H=\overset{N}{\underset{i=1}{\sum }}s_{i}^{2}$$

where *H* is the HHI, and s*_i_* is the proportion of groups in occupation *i*. For example, if all graduates from public health were distributed across two occupations equally: *H* = 0.5^2^ + 0.5^2^ = 0.5. However, if 90% of graduates entered one occupation, and 10% entered another: *H* = 0.9^2^ + 0.1^2^ = 0.82. Therefore, a higher HHI implies that more graduates are concentrated in fewer occupations. A lower HHI implies that the concentration of graduates are distributed more equally among the set of occupations.

### 2.4. Quantifying Mismatch

Mismatch can be generally defined as an employment scenario where an individual’s occupation does not align well with that individual’s qualification or field of study [23]. Mismatch is often known by other terms, such as overeducation, overqualification and underemployment, but more recent Australian literature tends to use the term mismatch [24]. From 2008 to 2015, the GDS captured information about the importance of study to graduates’ occupation. The GDS asked three questions regarding mismatch: “How important is the qualification you have just completed to your employment in your main paid job?”, “How important are the major fields of education you studied to your employment in your main paid job?”, and “How important are the other skills and knowledge acquired during your course to your employment in your main paid job?” For each of these questions, graduates could give one of the five following answers: formal requirement, important, somewhat important, not important and “do not know”. To answer research question 2, we classified an occupation as mismatched on the basis that a graduate answered either ‘not important’ or ‘do not know’ to these questions. We assigned a 1 to these answers, and a 0 to any other answer. We also assigned a 0 for no answer, reducing bias of assigning mismatch in the cohort. We then calculated the proportion of students mismatched as the sum of these values over the number of graduates. For example, if 4 students answered ‘do not know’ or ‘not important’ out of 10, then 0.4 or 40% were mismatched. Note, we considered each type of mismatch separately and represent them separately in our results sections.

### 2.5. How Well Does a Field of Education Prepare Graduates for an Occupation?

In our third research question, we examined how well public health graduates were ‘prepared’ for their occupations. Here, we defined being ‘prepared’ by not being mismatched (as per the methods above). In order to quantify how well prepared graduates were, we performed the following steps: Firstly, we iterated through each occupation; for each occupation, we noted which field of study produced the graduates with the lowest proportion of mismatch for that occupation; finally, we tallied these results and presented them as counts. For example, if the count for public health graduates equaled ten, this would mean there were ten occupations where public health graduates had the lowest proportion of mismatch out of all fields of education.

We represent our findings in two ways. Firstly, we present a table showing the occupations where public health graduates had the lowest proportion of mismatched students. This table aggregates all levels of education. Aggregating all levels of education assumes that there is only one set of occupational categories which all graduates are prepared for. This is likely an underestimate of the actual number of occupational categories (e.g., a program officer might have different positions for undergraduate and postgraduates, which are both coded as “Project or Program Officer”). Secondly, we compare public health cohorts to all other cohorts with level of education disaggregated and represent this using a scatter plot. In this plot, each point represents a field of education, the X-coordinate corresponds to the occupation HHI (as above), and the Y-coordinate corresponds to the number of occupations. Disaggregating by level of education assumes that there are four levels of occupational categories, one for each level of education. This is likely an overestimate of the number of occupational categories (e.g., it is unlikely that program officer positions are made distinct for Graduate Diploma, Master’s, Bachelor and PhD levels). We provided both sets of analyses as we felt both enable a better approximation of the actual number of occupational categories.

### 2.6. Data Analysis

All data analysis was performed in a Jupyter Lab environment using Python version 3.7.4. Numerical packages used for analysis were Pandas and Numpy, and Plotly was used for generating plots.

## 3. Results

### 3.1. Description of Graduate Cohorts

Table 1 presents a description of the graduate cohorts that are considered throughout the rest of this analyses. The cohorts of public health graduates tend to be older and have a higher representation of females and domestic students than students at the same level across all fields of education. In terms of employment statistics at the time of the survey, public health graduates had a lower unemployment rate, and tended to have a higher rate of full-time employment, except for graduates with a Bachelor’s degree and graduates with a PhD or Doctorate.

### 3.2. What Occupations Do Graduates Have?

#### 3.2.1. What Are the Most Common Occupations That Graduates of Public Health Have?

Table 2 presents the ten most common occupations that graduates have at the time they were surveyed (approximately four–six months after graduation). For Postgraduate Certificate or Diploma graduates, or Master’s by Coursework graduates, the most common occupations were ‘midwifery and nursing professionals’, and ‘medical practitioners’. These were substantially more common than other occupations. For example, ‘midwifery and nursing professionals’ was 10-foldmore common an occupation than ‘program or project administrator’ for Postgraduate Diploma and Certificates, and nearly 3-fold more common for Master’s by Coursework, despite being the third most common occupation. For these postgraduate levels, non-clinical positions included ‘health promotion officer’, ‘university lecturer’, ‘policy analyst’, ‘laboratory scientist’ and ‘health and welfare service managers’.

The most common occupation for Bachelor’s graduates was ‘sales assistant’, and other common occupations included clinical degrees such as ‘physiotherapist’, ‘occupational therapist’ and ‘podiatrist’. Non-clinical health occupations which were common included ‘health promotion officer’ and ‘program or project administrator’.

Doctoral and PhD graduates commonly held non-clinical occupations including ‘university lecturer’, ‘statistician’, ‘laboratory scientist’ and ‘health promotion officer’. The only clinical occupation which was commonly held by such graduates was ‘medical practitioner’.

#### 3.2.2. Is this Set of Occupations More or Less Heterogeneous Compared to Other Fields of Study (e.g., Business, Engineering, Humanities)?

**Figure 1:**
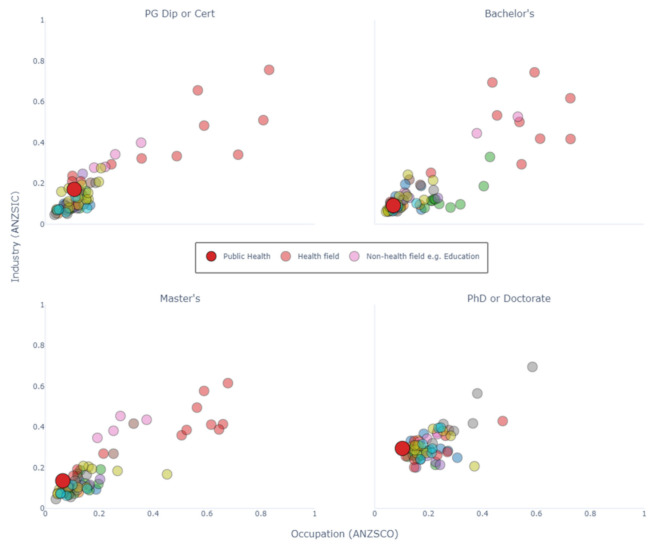
HHI showing concentration of occupation and industry by fields of education. Each panel represents graduates in a different level of education. Higher values indicate that graduates from the field of education are more concentrated in fewer occupations/industries. ANZSIC: Australian and New Zealand Standard Industrial Classification. ANZSCO: Australian and New Zealand Standard Classification of Occupations. An interactive version with labelled fields of education can be found in Supplementary Figure S1.

Figure 1 shows a scatter plot where each point represents the graduates from a field of study in higher education. The X-coordinate is the HHI for occupation, and the Y-coordinate is the HHI for industry. Points which are closer to the bottom-left corner are fields where few graduates go into the same occupation and industry. Three of four public health levels (Bachelor’s degree, Postgraduate Diploma or Certificate, and Master’s by coursework) are very close to the bottom-left corner, as are many fields of study such as ‘Business and Management’ and ‘Visual Arts’. This indicates that public health graduates are not concentrated in any one occupation or industry. Compared with other health fields (the smaller red circles), this is uncommon; the majority of health fields have a high HHI in both industry and occupation at all levels of education. The only two exceptions to this are public health and complementary therapies.

The one exception is the public health cohort from the PhD and Doctorate by coursework courses, which has an industry HHI closer to the median (the median industry HHI is substantially higher for this level overall), but a low occupational HHI compared with other fields. This implies that public health graduates with a PhD or Doctorate by coursework go into different occupations, despite entering similar industries.

### 3.3. Do Graduates Feel Mismatched to Their Occupations?

#### 3.3.1. To What Occupations Are Graduates Least Mismatched?

**Figure 2:**
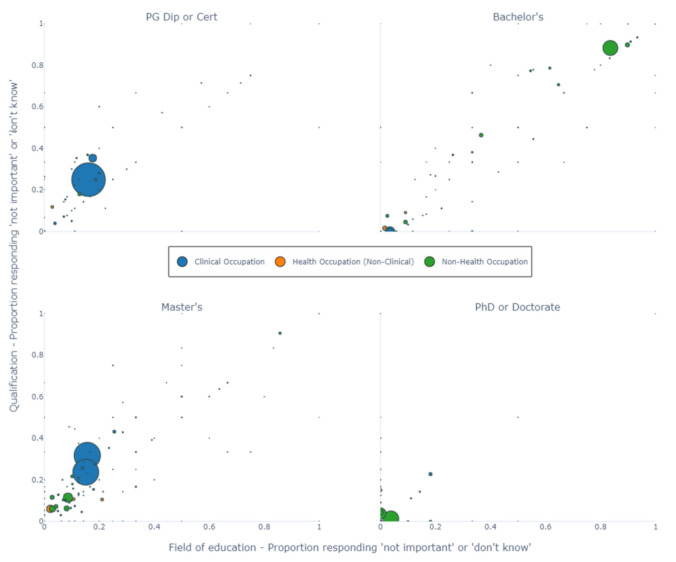
Proportion of public health graduates mismatched by occupation. Each panel represents graduates from a different level of education. *x*-axis is the proportion mismatched by field of education, *y*-axis is the proportion mismatched by qualification. Each circle represents graduates in a specific occupation. Radii of the circles represents the relative number of graduates. PG: Post-graduate. An interactive version with labelled fields of education can be found in the Supplementary Figure S2.

Figure 2 shows scatter plots for public health graduates only, where each point represents an occupation and both axes represent a type of mismatch: mismatch to the field of education on the *X*-axis, and mismatch to the qualification on the *Y*-axis. In the bottom-left corner of the plot are the occupations with the lowest proportion of mismatch. This area is where most occupations lie, with the exception of Bachelor’s graduates, where much of the volume is split between high and low mismatch areas (here, the occupations with high mismatch refer to non-health positions such as ‘sales assistant’ and ‘waiter’).

For postgraduate Certificates or Diplomas and Master’s by Coursework graduates, the large blue circles refer to ‘midwifery and nursing professional’ and ‘medial practitioner’. For these clinical occupations, there is a higher level of mismatch than non-clinical occupations (e.g., ‘health promotion officer’) and non-health occupations (e.g., ‘policy analyst’). The majority of occupations held by PhD or Doctoral students had very low levels of mismatch. The three occupations with the largest mismatch were clinical occupations (‘midwifery and nursing professional’, ‘medical practitioner’ and ‘clinical psychologist’).

#### 3.3.2. What Proportion of Graduates Are Mismatched, and How Does This Compare to Other Fields of Study?

**Figure 3:**
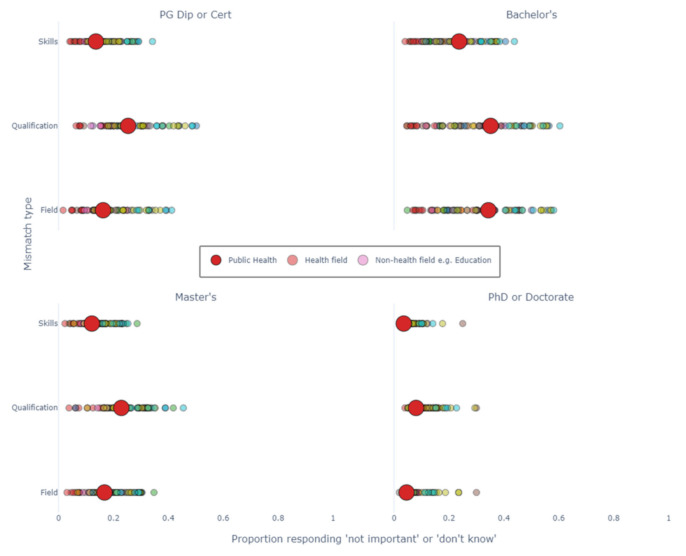
The proportion of graduates mismatched by field of study. Skills = proportion of graduates mismatched by residual skills. Qualification = proportion of graduates mismatched by their qualification. Field = proportion of graduates mismatched by field of education. PG: Post-graduate. An interactive version with labelled fields of education can be found in the Supplementary Figure S3.

Figure 3 plots the proportions of mismatched graduates (*x*-axis) by type of mismatch (*y*-axis), by field of study and level of education. These values are very closely correlated. Furthermore, the spread of the data is influenced by the level of education, with fields of education at the Bachelor’s level having the highest spread, and fields of education at the Doctoral/PhD level having the least spread.

For all educational levels except PhD and Doctorate, the proportion of mismatch for public health graduates tends to be around the midpoint of the data. For PhD and Doctorate graduates, the proportion of mismatch is much closer to the lowest point for all types of mismatch. When examining health fields (the red circles), public health graduates tend to have a higher proportion of mismatch than other fields, such as nursing or medicine.

Type of mismatch is highly correlated to other types of mismatch, but for all levels of education, cohorts report a lower level of skills mismatch than field or qualification mismatch. This is true of public health and in general. The inverse is true for qualification mismatch, which is higher on average for all levels of education, both for public health and other cohorts.

### 3.4. How Well Does Public Health Prepare Graduates for Their Occupation?

#### 3.4.1. Are There Occupations Where Public Health Graduates Have the Lowest Mismatch?

Table 3 presents the occupations for which public health graduates (aggregated by course level) have the lowest proportion of mismatch, compared with all other fields of study. Overall, there were seven occupations where the public health cohort had the least mismatch of any field of education. These occupations included professional and managerial occupations, which ranged between 2% and 7% of graduates reporting mismatch between their field of study and their occupation. These occupations included ‘primary health organisation manager’, and ‘health promotion officer’, as well as broader categorizations, such as ‘professionals nfd (not further defined)’ or ‘health and welfare services managers nec (not elsewhere classified)’.

#### 3.4.2. How Does This Compare to Other Fields of Study?

**Figure 4:**
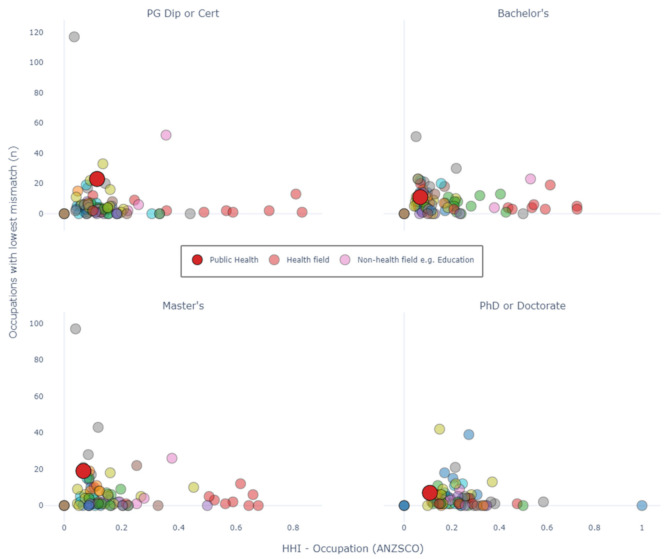
Number of occupations with the least mismatched graduates compared with all other fields of education. Circles represents graduates from a field of study (e.g., public health). X-coordinate = HHI for occupation. Y-coordinate = number of occupations that had the smallest proportion of mismatch, when that field of study was compared to all other fields of study. HHI, Herfindahl–Hirschman Index. NB, points at (0,0) indicate comparisons where there were zero graduates in the cohort. PG: Post-graduate. An interactive version with coded fields of education can be found in the Supplementary Figure S4.

Figure 4 presents scatter plots which show how common it is to produce graduates who are the least mismatched to an occupation, relative to an HHI value for occupation. To explain this clearly, consider the large red circle in the plot for “PG Dip or Cert”. This red dot represents the graduates for public health at the level of Postgraduate Certificate or Diploma. The X-coordinate is the HHI for occupation, which we have seen before in Figure 1. The Y-coordinate is the value 23. This value means that public health graduates reported the lowest average mismatch for 23 occupations, relative to all other fields of education at that same level of education.

For Postgraduate Diplomas or Certificates, and for Master’s by Coursework, public health graduates report the lowest average mismatch for 23 and 19 occupations, respectively. For Postgraduate Diplomas and Certificates, there are only three other fields of education which have higher values than this: Business and Management (117 occupations), Teacher Education (52 occupations) and Law (33 occupations). Comparatively, public health graduates from a Bachelor’s degree are least mismatched to a similar number of occupations as many other fields of study with a similar HHI (11 occupations). PhD and Doctoral students report a relatively low number of least mismatched occupations (7). However, for HHI indices between 0 and 0.2, only seven other fields of study report a higher value than this.

## 4. Discussion

We used eight years of graduate survey data to better understand the relationship between public health graduates and the occupations they have when they graduate. To our knowledge, this is the first study to quantify how mismatched public health graduates are to the occupations they have. This is also the first study to compare these results to other fields of education, in terms of the proportion of graduates who are mismatched. At present, if one searches for ‘public health career paths’, the information returned is typically information brochures from universities, explaining that students can go into a variety of occupations and industries and will list typical areas such as “occupational health and safety”, “health promotion”, “analyst”, and “epidemiology”. Our findings reinforce this: graduates from public health go into a variety of occupations and industries, and common occupations include health promotion officers and project administrators.

However, for graduates of public health, no occupation or industry is particularly common. Public health in this sense, is unlike vocational health degrees such as medicine or nursing, where the majority of graduates go into the same occupation and industry after graduation. Instead, the field of public health is more similar to business and management degrees in this respect. Therefore, saying that there are common occupations after studying public health is true, but misleading, as students may be assuming public health degrees give the same level of occupational certainty as medicine, nursing, or allied health. This is not the case.

Despite public health graduates going into a variety of occupations, it appears that they find themselves well prepared for occupations. This is true in an absolute sense (on average, less than one in five graduates feels mismatched) and in a relative sense (public health cohorts report a level of mismatch similar to the average mismatch of other fields, or less than this). In this respect, public health is similar to business and management degrees, which seem to balance the trade-off between occupational variance and mismatch well. This seems a notable achievement and may reflect the choices in public health schools regarding a curriculum which is both relevant and broad, or the versatility of graduates in finding employment. There are areas with greater mismatch, and this is useful for students to know. For example, there is a greater degree of mismatch in public health degrees at the Bachelor’s level than other levels. This may be expected, as undergraduates and postgraduates competing for the same positions should tend towards postgraduates being awarded the position ceteris paribus. Therefore, this may be appropriate if this is understood by both educators and students.

Can we consider occupations with low mismatch as ‘public health’ occupations? There seem two good reasons to do so. Firstly, having few graduates who feel mismatched with an occupation, seems like a good indication that there is a relationship between what was taught, and what the person is now doing in that occupation. Considering the findings for high mismatch are intuitive (e.g., occupations such as ‘waiter’, ‘clerk’ and ‘sales assistant’ had a very high proportion of mismatched graduates), it seems sensible to assume the inverse is true too. Secondly, these results are concordant with previous findings. For example, some of the main job advertisement offerings for public health jobs in Watts et al. (2019) were project officers, health promotion jobs, policy analyst jobs, and clinical health jobs. These were all common jobs for public health graduates here, and this is additional evidence that these jobs should be considered to be performing public health functions.

Another observation is the apparent dual audience of public health degrees: the clinical audience and the non-clinical audience, or more precisely, those who have clinical occupations after graduation, and those who do not. This is not a new phenomenon and has been noted in previous Australian research. Rotem et al. found that approximately half of MPH students surveyed had a clinical background or clinical position [5]. However, our research highlights that those who have clinical occupations do not feel public health was as useful as those with non-clinical occupations, regardless of degree level. It is difficult to make a judgement about whether this is a serious issue or not, but it deserves more research. For example, consider a nurse who studied an MPH to signal to their employer that they wanted to advance their career. They may report that the field was not important for their occupation, but may still be grateful they studied the degree. Consider another example, where one doctor felt their degree was not important, another three regarded their degree as highly important, and use their knowledge every day for important decisions. Is this a trade-off that Schools of Public Health are happy to make? This evidence highlights a broader issue that may impact Schools of Public Health: students may have very different reasons for undertaking the same degree. Considering this, further research into student motivations may be particularly helpful.

It is difficult to provide a direct comparison between our research and other studies, due to differences in the data used, and how data were analyzed. However, we can make some broad comparisons. In Australia, Li and Awofeso commented that their results “indicate strong demand and positive employment prospects for public health graduates” [6]. We can add to this statement, saying that there are a diverse range of positive employment prospects which graduates might find themselves well matched to, given a public health education. In the United States, Plepys et al. (2021), when analysing the destinations of 53,463 public health graduates, noted that public health graduates enter a variety of sectors, with government being a fairly moderate contributed of employment (17% of employed graduates enter government after graduation) [9]. This is in contrast to higher levels of government employment in Canadian [12] and South African [11] studies and indeed previous Australian findings [6]. The level of heterogeneity in outcomes in Australia and the United States should invite policy makers to question the presumption that investment in public health education will lead to a commensurate rise in government public health workers. Plepys et al. clarify this well, saying “Graduates’ first-destination outcomes provide academia insight into changes in the job market” [9] and it seems, in both Australia and in research from the United States, that the expectation that most public health graduates enter government conflicts with recent findings.

### Limitations and Suggestions for Further Research

There are limitations to this work which warrant discussion. We were limited by using the ASCED classifications for fields of study. In some ways, this was advantageous, as it gave us a clear set of educational fields against which to compare our results, and many of these categories are intuitive, e.g., Business and Management, or Nursing. However, the codes we used for public health do not necessarily only include broad public health degrees such as the Master of Public Health, and this is something to acknowledge. For example, many of the common occupations that Bachelor’s public health students held were clinical occupations, and this would have meant these students had studied another degree to obtain these occupations. These may be a case of misclassification, or a double degree, or that several students had studied a clinical degree only to study an undergraduate public health degree after this. This lack of clarity is added to by the fact that Bachelor’s level was aggregated from four course levels, albeit Bachelors (Pass) Degree was the most common. These are important limitations in an otherwise robust, representative dataset of higher education graduations.

Another important consideration is that we have only looked at people who are employed in occupations. In 2014, Li and Awofeso found that public health postgraduates had an unemployment rate of 5% [6], and unfortunately this looks to have increased, with our Master’s by Coursework cohort (a similar definition to Li and Awofeso’s cohort) having an unemployment rate of 9%. Public health is not unique in this regard, with unemployment rates increasing by 1.6 percentage points for all Postgraduate Diploma and Certificate graduates, up to 4.9 percentage points for all Master’s by Coursework graduates (source: GDS, data not shown). It is important to do further work into the drivers of employment outcomes for public health graduates, and what factors lead a graduate to having a mismatched occupation, or no occupation at all, and whether these drivers are unique to public health, or a function of a broader issue.

We were also limited by the ANZSCO classification system, as many occupational categories of interest were only defined by exclusion, e.g., “professionals not further defined”. Furthermore, considering the tremendous effect that COVID-19 has had on the world, there has been renewed emphasis in tracking those who contribute to public health work. In order to better plan for this work in the future, we need to capture these data at a national level, and to do so requires having classifications which are commensurate with the work being performed. For instance, it seems particularly important to understand the supply of graduates going into contact tracing occupations and epidemiology occupations, yet there exist no suitably granular classifications in the ANZSCO to capture these. We would recommend the addition of contact tracing and epidemiologist as a bare minimum, so that we can better monitor these positions in the future.

Finally, we also had limited information about the prior education and occupations of graduates. Firstly, as we did not know the prior education of graduates, we could only infer a subset of graduates who must have had a clinical education before undertaking public health education, as they were working in an accredited occupation (e.g., public health graduates with nursing occupations). This limited how we could disaggregate the data, such as a division between public health graduates who had previously received a clinical education, and those who had not. Such a division would better clarify questions around the dual audience of public health, and whether clinical graduates were more likely to stay in clinical occupations, or indeed a subset of them were using public health as an opportunity to change occupations.

## 5. Conclusions

Public health graduates find themselves in a heterogeneous set of occupations. Yet, despite this, graduates tend to report their field education, qualification, and acquired skills as relevant. This is an impressive balance between occupational heterogeneity and occupational mismatch and this finding seems to be especially true with more advanced levels of education, and when graduates have occupations which are non-clinical. However, this means that efforts to increase positions in governmental public health workforces may have the opportunity cost of displacing well-matched people in a broad variety of occupations and industries. As this research is exploratory, there would be benefit in further quantifying the drivers of mismatch in terms of education, occupation, salary, and demographics, as well as exploring differences between graduates with clinical and non-clinical backgrounds. Furthermore, the examination of mismatch over time, incorporating more recent survey data, would be of benefit.

## Figures and Tables

**Table 1:** Description of all public health graduate cohorts, compared with the same statistics when including all fields of education.

	Statistic	Bachelor’s Degree	Postgraduate Diploma or Certificate	Master’s by Coursework	PhD or Doctorate
**Public health graduates**	Count (n)	2346	1642	4336	588
	Age (median)	23	36	33	40
	Female (%)	80	74	72	72
	Domestic Student (%)	95	91	74	81
	Employed Full time (%)	42	61	56	62
	Employed Part time (%)	38	24	22	23
	Unemployed, seeking work (%)	8	4	9	6
	Not in the labour force (%)	11	10	13	9
**All other graduates**	Count (n)	636,144	134,323	223,406	31,875
	Age (median)	23	33	29	35
	Female (%)	61	65	54	52
	Domestic Student (%)	84	90	60	76
	Employed Full time (%)	46	60	53	63
	Employed Part time (%)	30	25	20	21
	Unemployed, seeking work (%)	10	6	14	7
	Not in the labour force (%)	14	9	13	9

**Table 2:** Ten most common occupations for all public health graduates by level of education. *nec, not elsewhere classified. **nfd, not further defined.

Course Level	Occupation (%)
Postgraduate Diploma or Certificate	Midwifery and Nursing Professionals—30.0%
	Medical Practitioners—7.0%
	Program or Project Administrator—3.0%
	Health Promotion Officer—2.0%
	Intensive Care Ambulance Paramedic—2.0%
	Occupational Therapist—2.0%
	Occupational Health and Safety Adviser—2.0%
	Physiotherapist—2.0%
	Social Worker—1.0%
	University Lecturer—1.0%
Bachelor’s Degree	Sales Assistant (General)—11.0%
	Physiotherapist—7.0%
	Occupational Therapist—5.0%
	Health Promotion Officer—3.0%
	Waiter—3.0%
	Program or Project Administrator—3.0%
	General Clerk—3.0%
	Podiatrist—2.0%
	Health Information Manager—2.0%
	Occupational Health and Safety Adviser—2.0%
Masters by Coursework	Medical Practitioners—13.0%
	Midwifery and Nursing Professionals—13.0%
	Program or Project Administrator—5.0%
	Health Promotion Officer—4.0%
	Professionals nfd—3.0%
	Policy Analyst—3.0%
	University Lecturer—2.0%
	Health and Welfare Services Managers nec*—2.0%
	Physiotherapist—2.0%
	Medical Laboratory Scientist—1.0%
Doctoral Degree or PhD	University Lecturer—18.0%
	Professionals nfd—12.0%
	Statistician—6.0%
	Medical Practitioners—4.0%
	Medical Laboratory Scientist—3.0%
	Health Promotion Officer—3.0%
	Health Professionals nfd**—3.0%
	University Lecturers and Tutors nfd**—3.0%
	Program or Project Administrator—3.0%
	University Tutor—2.0%

**Table 3:** The occupations for which public health graduates have the lowest proportion of mismatch, when compared to all other fields of study. *nfd: not further defined. **nec: not elsewhere classified. ANZSCO: Australian and New Zealand Standard Classification of Occupations.

Occupation (ANZSCO Description)	Proportion of Cohort Mismatched (Public Health)	Proportion of Cohort Mismatched (All Other Fields)
Primary Health Organisation Manager	0.02	0.08
Health Promotion Officer	0.02	0.08
Professionals nfd*	0.03	0.12
Occupational and Environmental Health Professionals nfd*	0.04	0.08
Other Health Diagnostic and Promotion Professionals nfd*	0.04	0.07
Health and Welfare Services Managers nec**	0.04	0.11
Health Diagnostic and Promotion Professionals nec**	0.07	0.1

## Data Availability

Availability of the Graduate Destinations Survey and the Graduate Outcome Survey should be sought by contacting Quality Indicators for Learning and Teaching (https://www.qilt.edu.au/qilt-surveys/graduate-employment, accessed on 1 January 2021]).

## Supplementary Materials

The following are available online at https://www.mdpi.com/article/10.3390/ijerph18147504/s1, Table S1: All occupations held by public health graduates, including data on proportion mismatched, and number of graduates with this occupation. Figure S1: Figure 1, interactive HTML version. Figure S2: Figure 2, interactive HTML version. Figure S3: Figure 3, interactive HTML version. Figure S4: Figure 4, interactive HTML version.
